# Linc-ROR induces epithelial-to-mesenchymal transition in ovarian cancer by increasing Wnt/β-catenin signaling

**DOI:** 10.18632/oncotarget.19545

**Published:** 2017-07-25

**Authors:** Yanhui Lou, Huanhuan Jiang, Zhumei Cui, Lingzhi Wang, Xiangyu Wang, Tian Tian

**Affiliations:** ^1^ Department of Gynecology, Affiliated Hospital of Qingdao University, Qingdao 266100, China

**Keywords:** ovarian cancer, long non-coding RNA, epithelial-to-mesenchymal transition, invasion, metastasis

## Abstract

Long intergenic non-protein coding RNA, regulator of reprogramming (linc-ROR) is an intergenic long non-coding RNA (lncRNA) previously shown to contribute to tumorigenesis in several malignancies. However, little is known about whether linc-ROR has a role in ovarian cancer progression. In this study, we found that linc-ROR expression was increased in high-grade ovarian serous cancer tissues compared with normal ovarian tissues or normal fallopian tube tissues. Furthermore, the level of linc-ROR expression was associated with ovarian cancer International Federation of Gynecology and Obstetrics stage and lymph node metastasis. Linc-ROR promoted ovarian cancer cell proliferation both *in vitro* and *in vivo*, and contributed to cell migration and invasion. Linc-ROR knockdown in ovarian cancer cell lines inhibited the epithelial-to-mesenchymal transition (EMT) program, which led to ovarian cancer cell metastasis through the repression of canonical Wnt/β-catenin signaling. Together, our results indicated that linc-ROR induces EMT in ovarian cancer cells and may be an important molecule in the invasion and metastasis of ovarian cancer.

## INTRODUCTION

Ovarian cancer has the highest mortality rate among all gynecological malignancies. Compared with early ovarian cancer, patients who are diagnosed with advanced ovarian cancer are more likely to have invasion and peritoneal metastasis to adjacent organs [1, 2]. High-grade ovarian serous cancer is a common type of ovarian cancer that is highly malignant and has an aggressive capacity; most affected patients die from their metastases [3]. Therefore, the genes associated with invasion and metastasis in ovarian cancer, especially in high-grade ovarian serous cancer, and their modulatory mechanisms have become attractive topics in recent years.

Previous studies have indicated that the epithelial-to-mesenchymal transition (EMT) is closely related to the invasion and metastasis of ovarian cancer [4]. The EMT program provides the molecular basis for the invasion and metastasis of epithelial tumor cells, and is a critical event in the early stages of invasion and metastasis of malignant tumors [5].

Long non-coding RNAs (lncRNAs) are a new group of RNAs that were recently discovered by the rapid development of sequencing techniques [6]. LncRNAs are over 200 nt in length, with no protein coding function, and are involved in transcriptional, post-transcriptional, and epigenetic gene regulation [7–9]. Recent studies revealed that lncRNAs regulate diverse cellular processes including stem cell pluripotency, differentiation, tumorigenesis, apoptosis, and metastasis [10–14]. Many lncRNAs, such as HOTAIR, H19, MEG3, and UCA1, have been recognized as key regulators of the occurrence and development of ovarian cancer [15–18]. Long intergenic non-protein coding RNA, regulator of reprogramming (linc-ROR) was first identified as a highly expressed transcript in induced pluripotent stem cells and embryonic stem cells that is regulated by the key pluripotency factors Oct4, Sox2, and Nanog [19, 20]. Linc-ROR has also been associated with tumorigenesis and EMT in many malignancies including breast, liver, pancreatic, and colon cancers [21–24]. However, the relationship between linc-ROR and EMT in ovarian cancer progression and metastasis remains unknown.

In this study, we assessed linc-ROR expression in high-grade ovarian serous cancer tissues, normal ovarian tissues, and normal fallopian tube tissues, and further analyzed the relationship between the level of linc-ROR expression and ovarian cancer International Federation of Gynecology and Obstetrics (FIGO) stage and lymph node metastasis. We then investigated the functions of linc-ROR in ovarian cancer cell proliferation both *in vitro* and *in vivo*, and its roles in cell invasion and metastasis. We demonstrate that the linc-ROR-induced changes in EMT in ovarian cancer cell lines are the result of alterations in the canonical Wnt/β-catenin signaling pathway. This study explored a new regulatory mechanism for linc-ROR in ovarian cancer invasion and metastasis, and provides new evidence for the therapeutic and prognostic value of linc-ROR in ovarian cancer.

## RESULTS

### Linc-ROR is up-regulated in human ovarian cancer tissues

**Figure 1:**
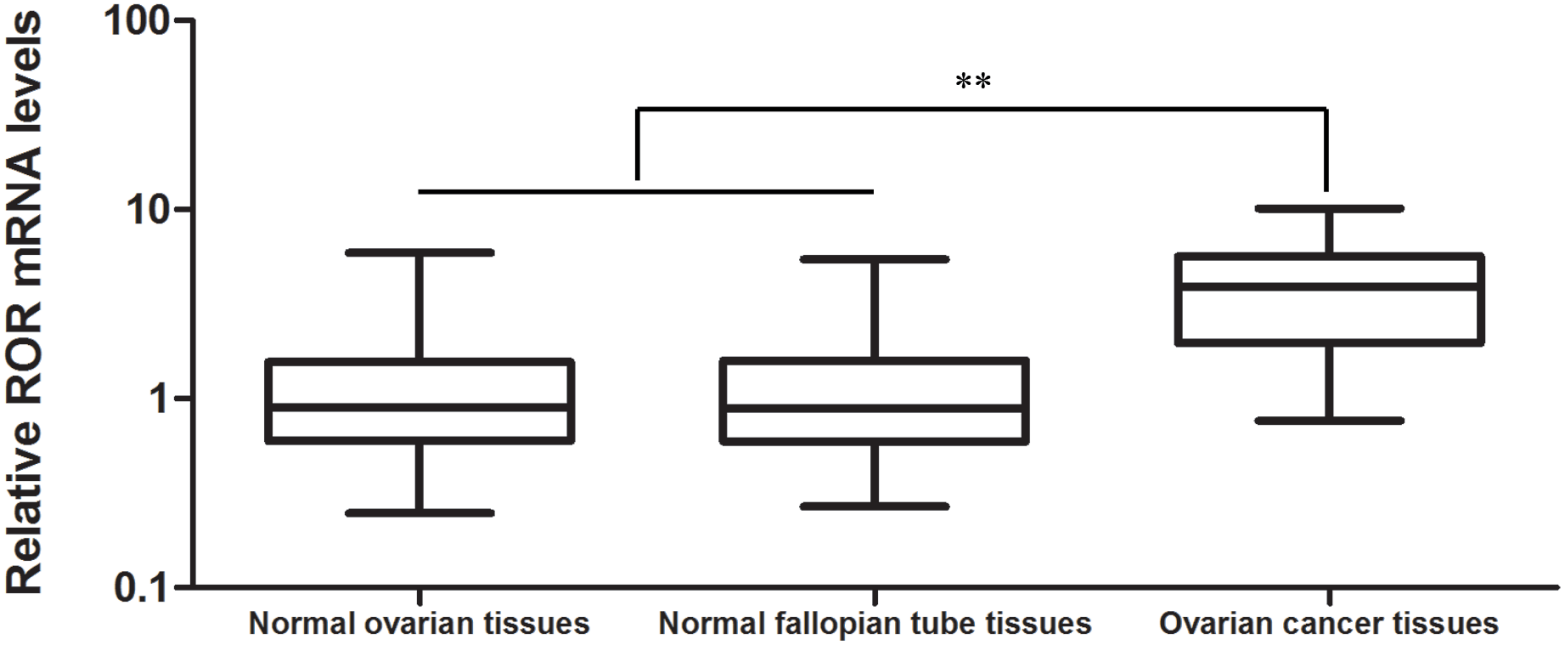
Linc-ROR mRNA expression by qRT-PCR in normal ovarian and fallopian tube tissues and high-grade ovarian serous cancer tissues The level of linc-ROR expression in high-grade ovarian serous cancer tissues was significantly higher than in normal ovarian or normal fallopian tube tissues. **P<0.01.

Linc-ROR mRNA expression was assessed by quantitative real-time (qRT)-PCR and shown to be significantly higher in the 39 high-grade ovarian serous cancer tissue samples than in either the 20 normal ovarian tissue samples or the 20 normal fallopian tube tissue samples (Figure 1, *P*<0.01). Table 1 shows the different levels of linc-ROR expression in patients subdivided by FIGO phases. Linc-ROR also showed a trend of increasing expression in patients with more advanced clinical phases of ovarian cancer (*P*<0.01), while patients with lymph node metastases had higher linc-ROR expression than those without (*P*<0.01). These results suggest that up-regulated linc-ROR expression may be associated with ovarian carcinogenesis and tumor progression.

**Table 1:** Linc-ROR expression in relation to clinic opathological parameters in high-grade ovarian serous cancer patients

Parameter	Cases (n)	Linc-ROR (mean±SD)	*P*-value
Age (years)			
<50	17	4.36±0.269	
≥50	22	4.44±0.327	0.432
FIGO stage			
I	5	3.79±0.0767	
II	15	4.11±0.0776	
III	13	4.44±0.136	
IV	6	5.06±0.256	<0.01
Residual tumor diameter (cm)			
<1	30	4.45±0.301	
≥1	9	4.78±0.253	0.163
Lymph node			
Negative	23	4.17±0.123	
Positive	16	4.74±0.315	<0.01
CA125 level (U/ml)			
<600	24	4.23±0.341	
≥600	15	4.64±0.449	0.259
Ascites			
<100	12	4.37±0.289	
≥100	27	4.57±0.367	0.674

### Linc-ROR promotes ovarian cancer cell proliferation *in vitro*

**Figure 2:**
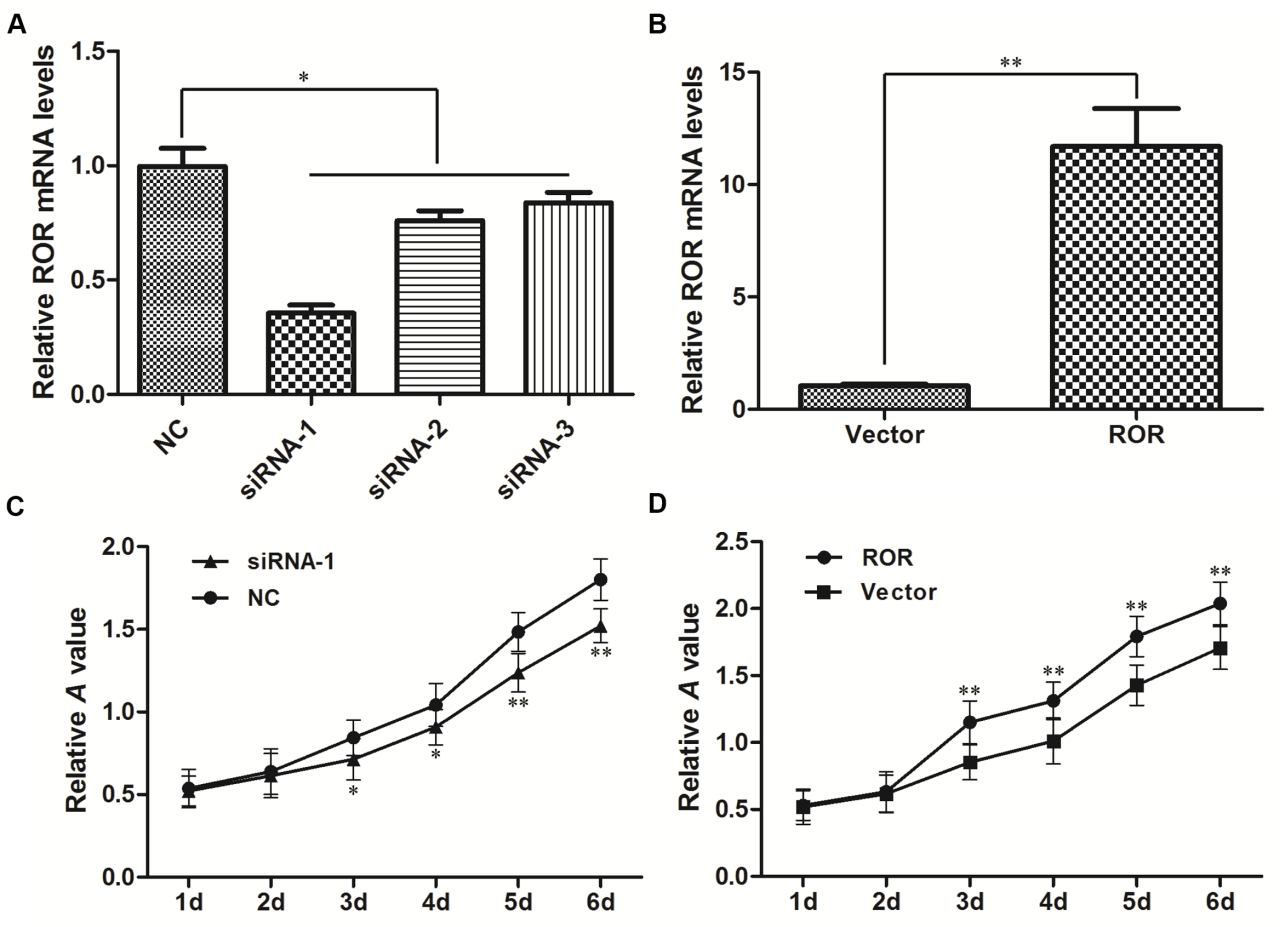
Linc-ROR mRNA expression in SKOV3 cells and its effects on proliferation qRT-PCR was used to detect linc-ROR expression after transfection with linc-ROR-targeting siRNA **(A)** or the linc-ROR expression plasmid **(B)**. GAPDH was used as an internal control. Linc-ROR knockdown reduced the proliferation rate of SKOV3 cells **(C)**. Linc-ROR overexpression promoted the proliferation rate of SKOV3 **(D)**. *P<0.05; **P<0.01.

To further investigate the effects of linc-ROR on the biological functions of ovarian cancer cells, we exogenously decreased linc-ROR expression by transfecting small interfering (si)-linc-ROR, and increased linc-ROR expression by transfecting the pIRES2-EGFP-linc-ROR vector. The levels of linc-ROR expression after transfection were confirmed by qRT-PCR. After siRNA transfection, linc-ROR expression was significantly lower in the si-linc-ROR groups than in the si-NC group (Figure 2A, *P*<0.05); linc-ROR expression was significantly higher after transfecting the linc-ROR expression plasmid than in cells transfected with empty vector (Figure 2B, *P*<0.01). siRNA-1 had the best inhibitory effect on linc-ROR expression, so we used it for subsequent experiments.

We then detected the proliferation rates of experimentally manipulated SKOV3 cells using the CCK-8 assay. Compared with the si-NC group, si-linc-ROR-transfected cells showed significantly reduced proliferation rates (Figure 2C). Conversely, linc-ROR overexpression significantly increased cell proliferation (Figure 2D). Similar results were obtained using A2780 cells (Supplementary Figure 1). Together, these results suggest that linc-ROR may be an important regulator of proliferation in ovarian cancer cells *in vitro*.

### Linc-ROR promotes migration and invasion in ovarian cancer cells

**Figure 3:**
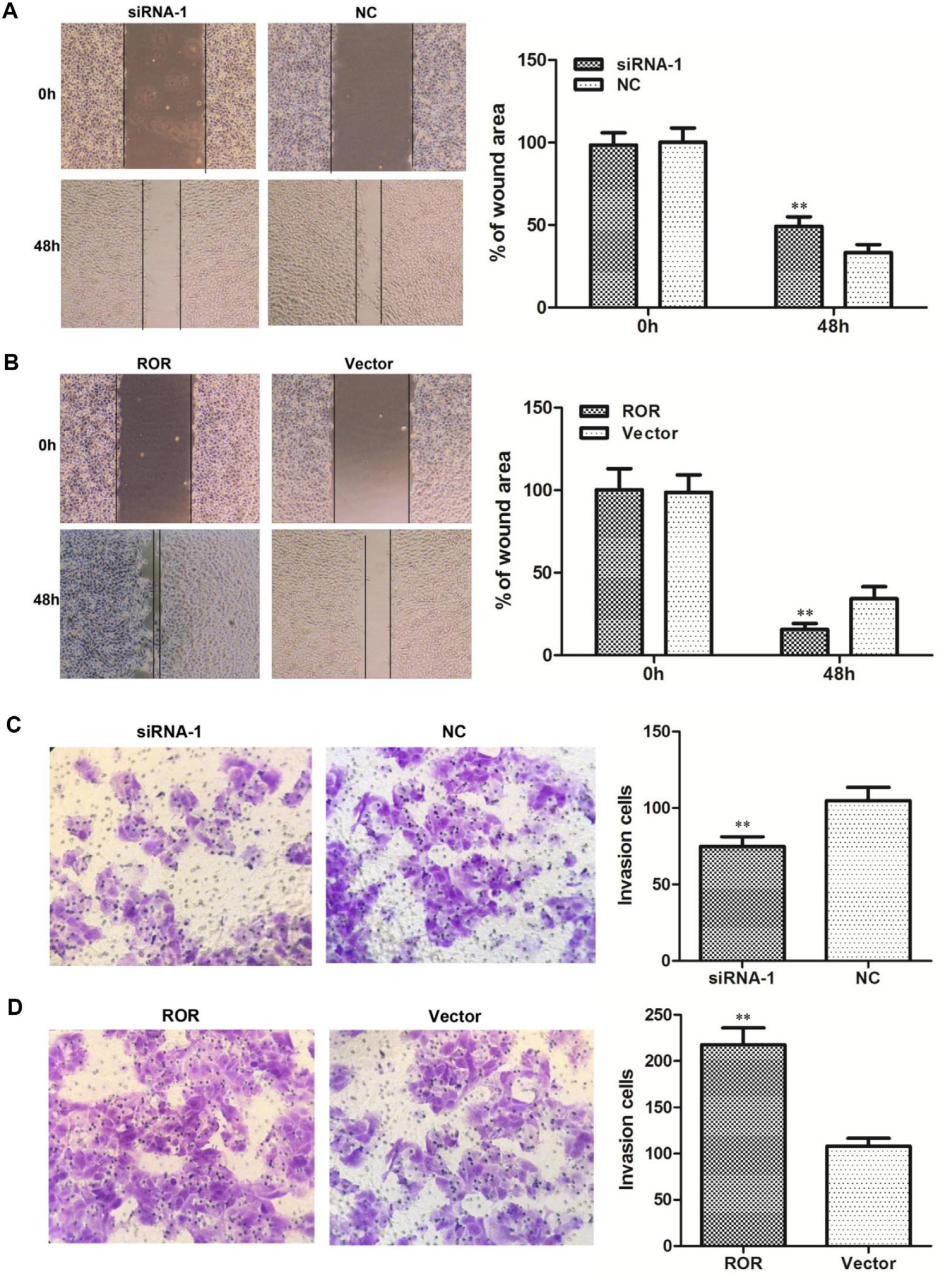
Effects of linc-ROR on the migration and invasion of SKOV3 cells Linc-ROR promoted the migratory and invasive abilities of ovarian cancer cells. Wound healing assay results **(A, B)**; transwell migration assay results **(C, D)**. **P<0.01.

We next investigated the biological effects of linc-ROR on the migration and invasion of ovarian cancer cells. Wound healing assays showed that linc-ROR knockdown reduced SKOV3 cell migration (Figure 3A), while linc-ROR overexpression increased wound healing (Figure 3B). Moreover, transwell assays revealed that reducing linc-ROR expression inhibited SKOV3 cell invasion (Figure 3C), while linc-ROR overexpression remarkably stimulated invasion (Figure 3D). Similar results were obtained using A2780 cells (Supplementary Figure 2). These results indicate that linc-ROR promotes the migratory and invasive abilities of ovarian cancer cells.

### Ectopic linc-ROR expression induces an EMT program in SKOV3 cells

**Figure 4:**
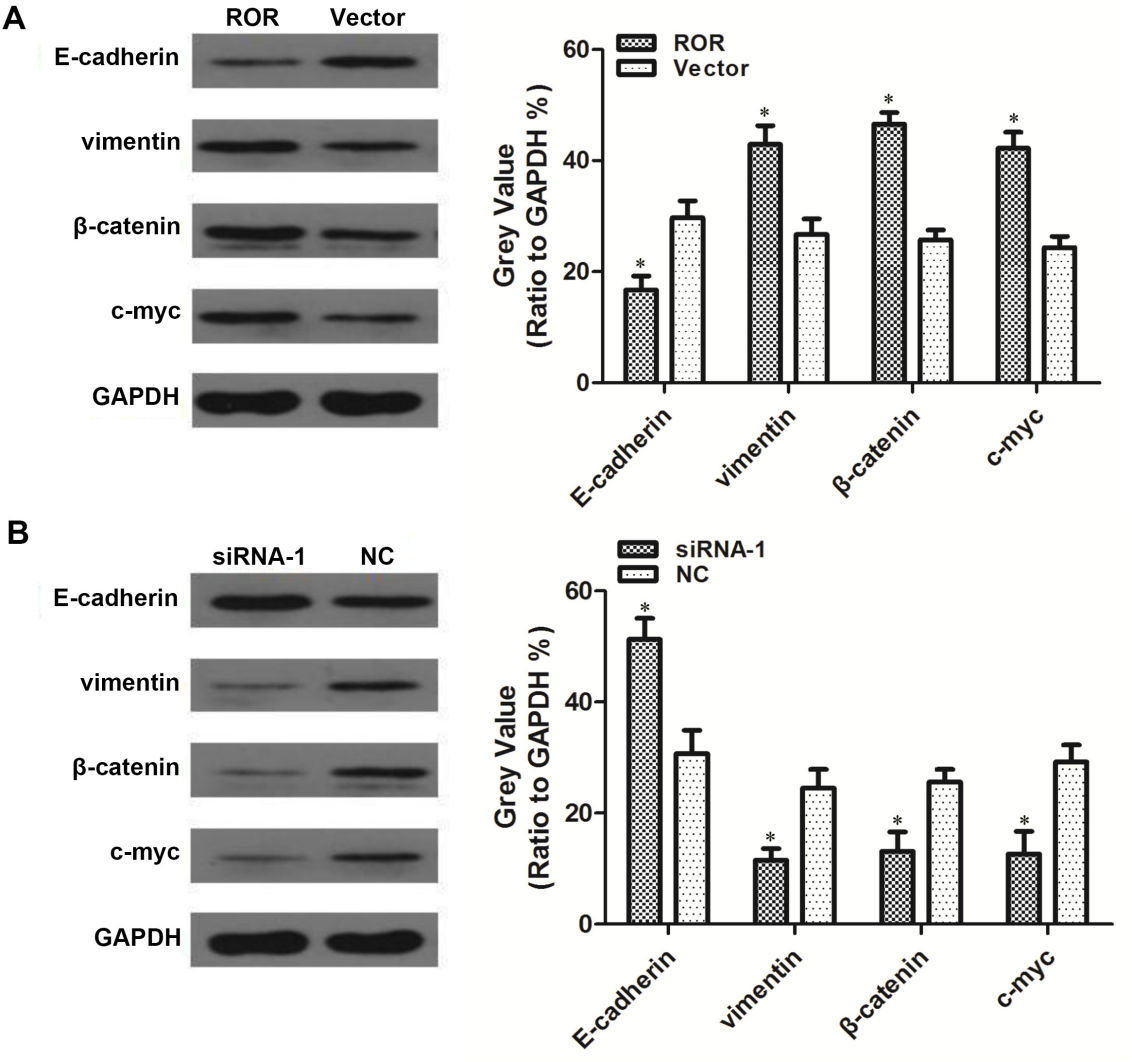
Linc-ROR enhances EMT in SKOV3 cells Western blotting revealed decreased E-cadherin expression in linc-ROR-overexpressing cells compared with cells in the empty vector group, whereas vimentin, β-catenin, and c-myc were increased in the experimental group compared with controls **(A)**. Higher E-cadherin expression was detected in the siRNA-1 group compared with the NC group, whereas vimentin, β-catenin, and c-myc were significantly decreased in the siRNA-1 group compared with the NC group **(B)**. *P<0.05.

To investigate the oncogenic role of linc-ROR in ovarian cancer EMT, we detected EMT marker expression via western blotting. Levels of the epithelial marker E-cadherin were reduced in SKOV3 cells exogenously overexpressing linc-ROR compared with cells transfected with empty vector. Accordingly, levels of the mesenchymal marker vimentin and the EMT-associated proteins β-catenin and c-myc were increased in linc-ROR-overexpressing SKOV3 cells compared with cells carrying empty vector (Figure 4A). Conversely, E-cadherin expression was higher in linc-ROR-silenced SKOV3 cells than in the control group, whereas vimentin, β-catenin, and c-myc were prominently decreased in linc-ROR-silenced SKOV3 cells compared with controls (Figure 4B). These results suggest that linc-ROR promotes EMT in SKOV3 cells.

### Linc-ROR promotes ovarian cancer tumor growth *in vivo*

**Figure 5:**
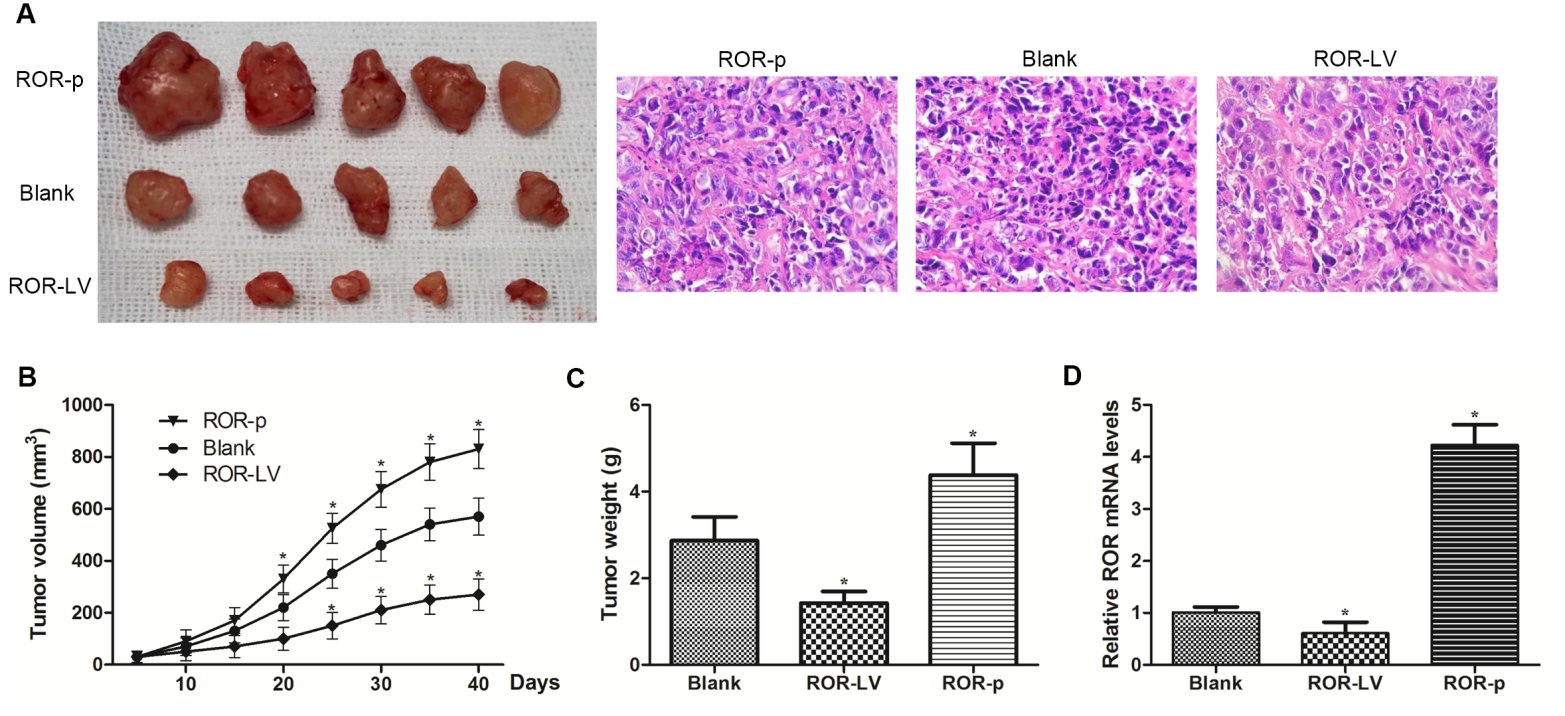
Linc-ROR promotes tumor growth in nude mice **(A)** Xenograft tumors were removed from nude mice and histological changes were shown (×400). **(B)** ROR-p cell-derived xenograft tumors grew more quickly compared with ROR-LV and control SKOV3 cell-derived tumors. **(C)** The mean weight of ROR-p cell-derived xenograft tumors was significantly larger than ROR-LV and control SKOV3 cell-derived tumors. **(D)** The expression level of linc-ROR in samples of xenograft tumor.

The EMT process has been implicated in ovarian cancer metastasis. To determine whether linc- ROR could regulate tumorigenesis of ovarian cancer cells *in vivo*, we performed assays in immunodeficient mice. SKOV3-ROR-LV, SKOV3-ROR-p, and control SKOV3 cells were inoculated to establish a subcutaneous xenograft model using nude mice. Compared with SKOV3-ROR-LV and control SKOV3 cells, mice inoculated with SKOV3-ROR-p cells formed larger xenograft tumors (Figure 5A–5C). By contrast, the SKOV3-ROR-LV cell group formed smaller tumors than the other groups. Assessment of linc-ROR expression in xenograft tumors by qRT-PCR showed that it was clearly increased in SKOV3-ROR-p group tumors compared with other groups (Figure 5D). This suggests that a high level of linc-ROR can promote tumor growth. Consequently, the *in vivo* results indicate that linc-ROR has a pivotal role in ovarian cancer tumor growth.

### Linc-ROR promotes EMT by upregulating the Wnt/β-catenin pathway

**Figure 6:**
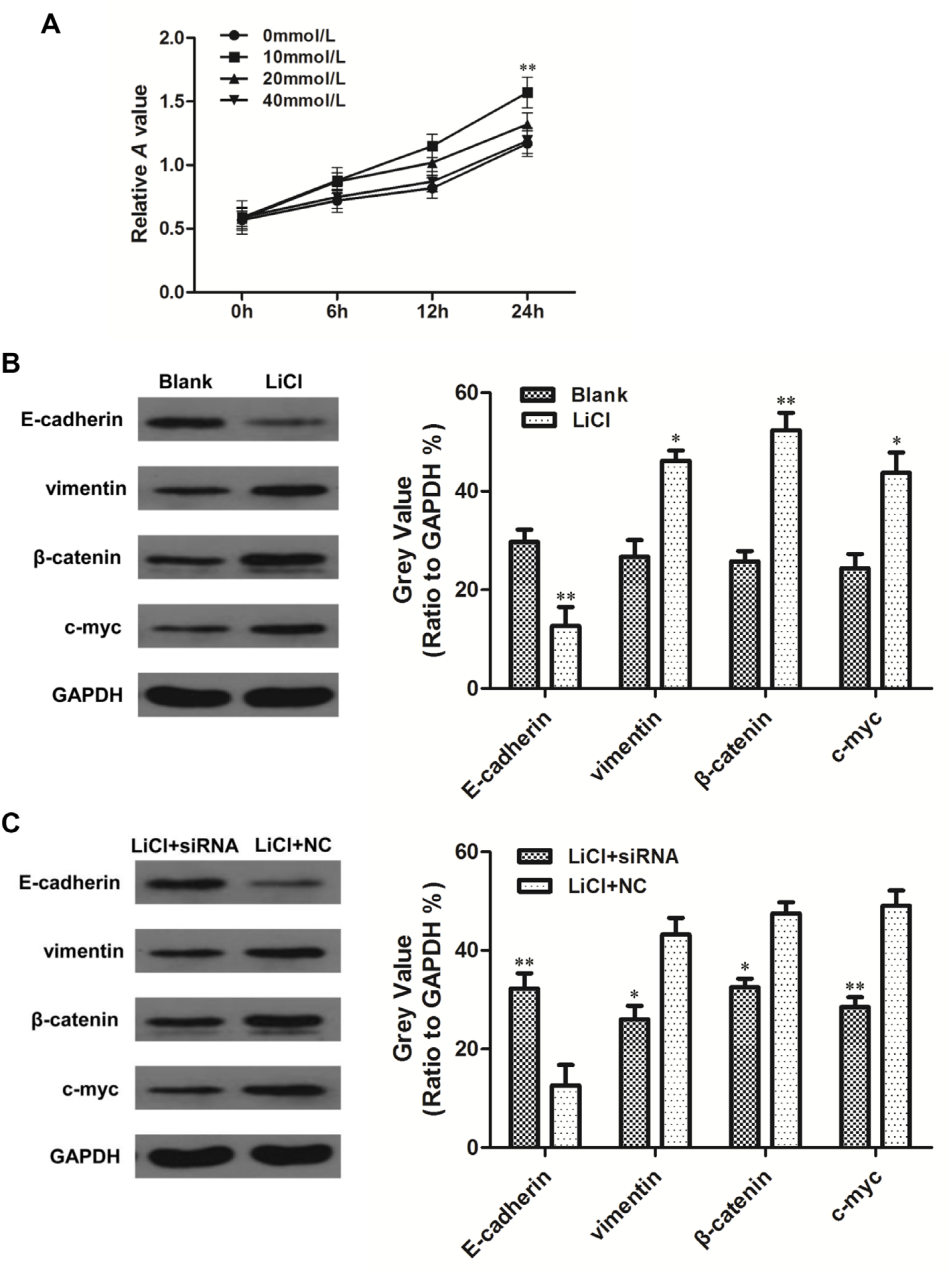
Linc-ROR promotes EMT in SKOV3 cells by activating the Wnt/β-catenin pathway SKOV3 cells were incubated with different concentrations of LiCl (0, 10, 20, and 40 mM), and after 6, 12, and 24 h, the CCK-8 assay was used to detect cell proliferation. Cell proliferation was most strongly promoted by 10 mM LiCl treatment for 24 h **(A)**. When treated with LiCl only, E-cadherin expression was decreased and vimentin, β-catenin, and c-myc were increased compared with controls **(B)**. When linc-ROR was silenced following LiCl treatment, E-cadherin expression was increased and vimentin, β-catenin, and c-myc were decreased compared with cells transfected with si-NC **(C)**. **P<0.01.

We have demonstrated that linc-ROR overexpression or silencing changed the level of β-catenin and c-myc expression (Figure 4). β-catenin is a key protein in the canonical Wnt/β-catenin pathway and forms adherens junctions with E-cadherin; c-myc is a Wnt/β-catenin pathway target gene. We hypothesized that linc-ROR may have triggered an EMT program in ovarian cancer cells by upregulating Wnt/β-catenin signaling. To independently determine the effect of Wnt/β-catenin signaling activation on SKOV3 cell proliferation and to determine the optimal LiCl (a Wnt/β-catenin pathway activator) dose to achieve this, we treated SKOV3 cells with different doses of LiCl for different lengths of time. Treatment with 24-h 10 mM LiCl promoted more proliferation than the other tested doses and treatment times (Figure 6A). Therefore, we used 24-h 10 mM LiCl in subsequent experiments. E-cadherin was decreased and vimentin, β-catenin, and c-myc were increased in 24-h 10 mM LiCl-treated SKOV3 cells compared with untreated controls (Figure 6B). Linc-ROR siRNA was then transfected into the cells, and we subsequently detected increased E-cadherin and decreased vimentin, β-catenin, and c-myc expression compared with the si-NC group (Figure 6C). These results demonstrate that even when the Wnt/β-catenin pathway is exogenously activated by LiCl, silencing linc-ROR expression can inhibit downstream effects of Wnt/β-catenin signaling. Therefore, we conclude that linc-ROR promotes ovarian cancer EMT at least in part by activating the Wnt/β-catenin pathway.

## DISCUSSION

Ovarian cancer is a malignant tumor that is dangerous to women’s health because it has the highest mortality rate among all malignancies of the female reproductive system. One important reason for this is the strong invasive and metastatic abilities of ovarian cancer cells. This study identified an oncogenic role for linc-ROR in ovarian cancer development and metastasis. Our results indicated that linc-ROR is highly expressed in high-grade ovarian serous cancer tissues, and that high linc-ROR expression is associated with advanced FIGO stage and lymph node metastasis. Moreover, exogenously upregulating linc-ROR expression in ovarian cancer cells promoted proliferation, migration, and invasion *in vitro*, and promoted tumor growth in immunodeficient mice *in vivo*. We also discovered that linc-ROR can induce EMT to promote invasion and metastasis, and that the Wnt/β-catenin pathway may activate linc-ROR-induced EMT. These results provide further evidence for the role of increased linc-ROR expression in malignant ovarian cancer phenotypes.

Linc-ROR is a non-protein coding RNA, approximately 2.6 kb in length that is located on chromosome 18q21.31. It was first identified in induced pluripotent stem cells, where it plays important roles in maintaining stem cell pluripotency [19]. It was reported to mainly be expressed in the cytoplasm where it suppresses p53 translation through direct interaction with the heterogeneous nuclear ribonucleoprotein I in response to DNA damage [25]. In recent years, accumulating evidence has revealed that linc-ROR is highly expressed in a variety of tumors, such as malignancies of the breast [21], liver [22], pancreas [23] and colon [24]. High linc-ROR expression is thought to enhance tumor growth, migration and invasion, promote EMT, and/or influence the characteristics of cancer stem cells. Hou et al. found that linc-ROR significantly enhanced the invasion and metastasis of breast cancer cells by acting as a molecular sponge for miR-205 [21]. Additional studies have indicated that linc-ROR regulates tumor stem cell differentiation, and promotes tumor cell proliferation, invasion and metastasis by regulating Oct4, Sox2, and Nanog expression via interfering with micro RNA-145 [23, 26, 27]. Therefore, linc-ROR appears to play a role in EMT programs, which promotes the invasive and metastatic abilities of tumor cells. Other lncRNAs have also been shown to be associated with ovarian cancer development and metastasis, including HOTAIR, H19, GAS5, and UCA1 [15–18]; however, to date, little was known about linc-ROR in ovarian cancer.

We first examined linc-ROR expression in samples of high-grade ovarian serous cancer tissue, normal ovarian tissue, and normal fallopian tube tissue. Linc-ROR mRNA was shown to be expressed at higher levels in high-grade ovarian serous cancer tissues than in normal ovarian or normal fallopian tube tissues. Analysis of suggested that patients with more advanced ovarian cancer (those with an advanced FIGO stage) have increased tumoral linc-ROR expression; furthermore, linc-ROR expression was higher in ovarian cancer tissues from patients with than without lymph node metastases. These results suggest that linc-ROR may be associated with the development and metastasis of ovarian cancer. To investigate the oncogenic role of linc-ROR in ovarian cancer, we exogenously silenced or overexpressed linc-ROR in ovarian cancer cells using siRNA, or lentivirus and expression plasmids. Ectopically expressed linc-ROR promoted proliferation *in vitro* and *in vivo*, contributed to cell migration and invasion, and inhibited the epithelial marker E-cadherin, while stimulating expression of the mesenchymal marker vimentin in an ovarian cancer cell line. We also detected upregulated β-catenin and c-myc in exogenously overexpressing linc-ROR cells compared with control cells, providing further evidence that linc-ROR induces EMT in ovarian cancer cells. These outcomes were reversed when linc-ROR was silenced by siRNA.

EMT is one of the most important steps of tumor metastasis and plays a central role in tumor progression. During this process, cells lose epithelial marker expression and instead express mesenchymal markers, which induces the loss of cell–cell and cell–matrix adhesion, and increases migratory, invasive, and stem cell properties [28]. EMT is essential for tumor invasion and metastasis, and embryonic development. Blechschmidt et al. found that E-cadherin and Snail expression were negatively correlated in ovarian cancer. The E-cadherin/Snail ratio could therefore reflect the extent of malignancy for ovarian cancer because E-cadherin and Snail expression is associated with tumor invasion and metastasis [29]. Gao et al. discovered that NAC1 was upregulated in ovarian cancer tissues, and that highly expressed NAC1 decreases nuclear factor-κB phosphorylation (induced by the metastasis suppressor gene *MKK4*) and promotes Twist expression, which then inhibits E-cadherin expression and induces EMT in ovarian cancer cells; in their experiments the invasive and metastatic abilities of ovarian cancer cells were accordingly enhanced [30]. In our study, we found that linc-ROR was associated with expression of the EMT-associated markers E-cadherin, vimentin, β-catenin, and c-myc in ovarian cancer cells. Therefore, linc-ROR may be a key regulator of ovarian cancer EMT and metastasis.

A variety of signaling pathways, including transforming growth factor-β, mitogen-activated protein kinase, Notch, and Wnt, can trigger EMT. The canonical Wnt/β-catenin pathway has been proven to induce EMT in ovarian cancer, breast cancer, and colon cancer cells [31]. When the Wnt/β-catenin pathway is abnormally activated, adhesion between tumor cells is weakened, which induces EMT and enhances the ability of tumor cells to invade and metastasize. Moreover, β-catenin is a key protein in the Wnt/β-catenin pathway that forms adherens junctions together with E-cadherin [32]. In our study, we found that β-catenin expression was altered in response to experimental changes in linc-ROR expression. Western blotting indicated that activation of the Wnt/β-catenin pathway led to a decrease in E-cadherin expression, and increased vimentin, β-catenin, and c-myc expression. However, following linc-ROR silencing, E-cadherin was increased and vimentin, β-catenin, and c-myc were decreased. This demonstrates that even in a forced state of Wnt/β-catenin pathway activation, silencing linc-ROR inhibits the downstream effects of Wnt/β-catenin signaling. Further experimentation confirmed that linc-ROR triggered ovarian cancer cells to undergo EMT through Wnt/β-catenin pathway activation. Thus, we conclude that linc-ROR mediates the invasion and metastasis of ovarian cancer cells at least in part by activating EMT through Wnt/β-catenin signaling.

In conclusion, this study demonstrated that linc-ROR is up-regulated in high-grade ovarian serous cancer tissues, where it promotes proliferation, invasion, and metastasis. Furthermore, we showed that linc-ROR acts as a potential oncogene in ovarian cancer by initiating an EMT program, and that the Wnt/β-catenin pathway may also be involved in this process. These results suggest that linc-ROR is a potential biomarker and therapeutic target for ovarian cancer.

## MATERIALS AND METHODS

### Ethics statement

This investigation has been conducted in accordance with the ethical standards and according to the Declaration of Helsinki and according to national and international guidelines and has been approved by the authors’ institutional review board. All experimental procedures were approved by the Institutional Review Board of the Ethics Board of the Affiliated Hospital of Qingdao University, and all subjects signed written informed consent forms.

### Patients and tissue specimens

Ovarian cancer tissue specimens were obtained from patients (average age, 55 years) who had undergone surgical treatment for ovarian cancer at the Affiliated Hospital of Qingdao University between March 2015 and March 2016. Normal ovarian tissue and normal fallopian tube tissue specimens came from patients (average age, 53 years) who had undergone surgical treatment for hysteromyoma at the Affiliated Hospital of Qingdao University in the same timeframe. All cases were confirmed by postoperative pathological diagnosis. Patients who had received neoadjuvant chemotherapy or radiation therapy before surgery were excluded from this study. We collected 39 high-grade ovarian serous cancer tissue samples (in accordance with FIGO classification standards; five cases of phase I, 15 cases of phase II, 13 cases of phase III, and six cases of phase IV), 20 normal ovarian tissue samples, and 20 normal fallopian tube tissue samples. Linc-ROR mRNA expression was evaluated by qRT-PCR in all samples, which were stored at −80°C immediately after the operation.

### Cell lines

The human epithelial ovarian cancer cell lines SKOV3 and A2780 were purchased from the Chinese Academy of Science (Shanghai, China). Cells were cultured in RPMI-1640 medium (Hyclone, Logan City, UT, USA) or DMEM (Hyclone), both supplemented with 10% fetal bovine serum (FBS; Hyclone), 100 U/mL penicillin, and 100 mg/mL streptomycin in a humidified incubator at 37°C with 5% CO_2_.

### RNA extraction and qRT-PCR

Total RNA was extracted from tissue samples and cells on ice using TRIzol reagent (Invitrogen, Carlsbad, CA, USA) and reverse-transcribed into cDNA using the TRUEscript 1st Strand cDNA Synthesis Kit (Aidlab, Beijing, China) following the manufacturer’s protocol; reactions were incubated for 30 min at 42°C, 5 min at 85°C, and then stored at −20°C. qRT-PCR reactions were performed using 2×SYBR Green qPCR Mix (Aidlab) on an ABI 7900HT sequence detection machine (Thermo Fisher Scientific, Waltham, MA, USA); reactions were incubated at 95°C for 3 min, followed by 40 cycles of 95°C for 15 s and 60°C for 40 s. GAPDH was used as an internal control. The primer sequences were as follows: linc-ROR forward: 5'-GAAGGTTCAACATGGAAACTGG-3', and reverse: 5'-TGAGACCTGCTGATCCCATTC-3'; GAPDH forward: 5'-CTCAGACACCATGGGGAAGGTGA-3', and reverse: 5'-ATGATCTTGAGGCTGTTGTCATA-3'. Both target (linc-ROR) and reference (*GAPDH*) genes were amplified in separate wells and run in triplicate. Statistical analyses of the results were performed using the 2^−ΔΔCT^ relative quantification method.

### Plasmids and siRNA infection

The overexpression plasmid plRES2-EGFP-linc-ROR was designed, synthesized, and extracted by Youbio Biological Company (Changsha, China). Ovarian cancer cells cultured in 6-well plates were transfected with the plRES2-EGFP-linc-ROR or empty vector using Lipofectamine 2000 (Invitrogen) according to the manufacturer’s instructions. Linc-ROR knockdown was performed using siRNA purchased from RiboBio Co. (Guangzhou, China). Ovarian cancer cells cultured in 6-well plates were transfected with si-linc-ROR or siRNA-negative control (siNC) using riboFECT™ CP (RiboBio Co.) according to the manufacturer’s instructions. Cells were harvested 48 h after transfection for qRT-PCR to detect transfection efficiencies.

### Cell proliferation assay

Cell proliferation was monitored using the Cell Counting Kit-8 (CCK-8; Yiyuan Biotechnologies, Guangzhou, China). After 48-h transfection with the linc-ROR expression plasmid or si-linc-ROR, ovarian cancer cells were seeded into 96-well plates (3000 cells/well), and cell proliferation was documented every 24 h for 6 d following the manufacturer’s instructions. All experiments were performed in quadruplicate. The number of viable cells was assessed by measuring absorbance at 450 nm using an enzyme-linked immunometric meter (Thermo Fisher Scientific).

### Wound healing assay

After 48-h transfection with the linc-ROR expression plasmid or si-linc-ROR, ovarian cancer cells were seeded into 6-well plates (3×10^5^ cells/well). When the cells reached approximately 90% confluence, cell layers were wounded using a sterile 200-μL pipette tip and washed with phosphate-buffered saline to remove cell debris. The cells were then cultured in medium with 1% FBS. Scrape lines were photographed with a light microscope at the indicated time points. Each experiment was performed in triplicate.

### Transwell migration assay

Cell invasion was measured in Matrigel-coated (BD Biosciences, San Diego, CA, USA) transwell inserts (8-μm pore size; Corning, Corning, NY, USA). After 48-h transfection with the linc-ROR expression plasmid or si-linc-ROR, 5×10^4^ cells were placed into the upper chamber inserts in serum-free media, while the bottom chambers were filled with 750 μL of conditioned medium containing 20% FBS. After 48-h incubation, cells that did not penetrate through the Matrigel were removed with a cotton swab. Cells that invaded and adhered to the lower surface of the insert were fixed and stained with a methanol and 0.1% crystal violet solution, then photographed and counted using an inverted microscope. The number of invaded cells was determined in five random fields per chamber, and the mean value was calculated. All experiments were performed independently in triplicate.

### Western blot analysis

Cells were washed twice with PBS and lysed in RIPA buffer (Beyotime, Haimen, China) supplemented with protease inhibitor cocktail (Roche, Basel, Switzerland). Protein concentrations were measured using the BCA Protein Assay Kit (Beyotime). Protein lysates were subjected to 10% sodium dodecyl sulfate polyacrylamide gel electrophoresis, transferred to polyvinylidene fluoride membranes (Millipore, Billerica, MA, USA) and incubated with primary antibodies for 24 h at 4°C. Membranes were then washed and incubated with horseradish peroxidase-conjugated secondary antibodies for 1 h at room temperature, and immunoreactive bands were visualized with ECL chromogenic substrate (ComWin Biotech Co., Beijing, China). GAPDH was used as an internal control. Primary antibodies were as follows: E-cadherin (Ab133597; Abcam, Cambridge, UK), vimentin (CBL202; EMD Millipore, Billerica, MA, USA), β-catenin (Ab32572; Abcam), c-myc (Ab32072; Abcam), and GAPDH (ENM0215; Elabscience, Bethesda, MD, USA).

### *In vivo* tumor growth assays

Female BALB/c nude mice (4-weeks-old) were purchased from Slac Laboratory Animal Company (Shanghai, China). Lentivirus LV-LINC-ROR-RNAi (Shanghai Genechem Co., China) was transfected into SKOV3 cells to stably decrease the linc-ROR expression level, and plasmid plRES2-EGFP-linc-ROR was used to stably increase linc-ROR expression in SKOV3 cells. SKOV3-ROR-LV cells (transfected with lentivirus LV-LINC-ROR-RNAi), SKOV3-ROR-p cells (transfected with plasmid plRES2-EGFP-linc-ROR), and control SKOV3 cells (5×10^6^) were subcutaneously injected into the armpit of nude mice. The mice were killed 40 days after inoculation, and xenograft tumors were surgically isolated. Tumors were photographed and weighed later, collected to stain with hematoxylin and eosin for histological analysis and the expression of linc-ROR was assessed by qRT-PCR. All animal experiments were approved by the Animal Care Committee Affiliated Hospital of Qingdao University.

### Statistical analysis

SPSS 17.0 (SPSS, Chicago, IL, USA) and GraphPad Prism 5.0 (GraphPad Software, La Jolla, CA, USA) were used for statistical analysis and the graphical presentation of data. Data are presented as means±SD. The statistical significance of the results was analyzed by fold change and the Student’s *t*-test. Differences were considered statistically significant at *P*<0.05.

## SUPPLEMENTARY MATERIALS FIGURES



## Abbreviations

Linc-ROR: long intergenic non-protein coding RNA-ROR
LncRNA: long non-coding RNA
EMT: epithelial-to-mesenchymal transition
FIGO: International Federation of Gynecology and Obstetrics
siRNA: small interference RNA
NC: negative control
qRT-PCR: real-time reverse transcription-PCR
CCK-8: Cell Counting Kit-8
LiCl: lithium chloride
