# Supplementary material for: Linc-ROR induces epithelial-to-mesenchymal transition in ovarian cancer by increasing Wnt/β-catenin signaling
Source: Oncotarget. 2017 Jul 25;8(41):69983–94. doi: 10.18632/oncotarget.19545 (PMC5642532; doi:10.18632/oncotarget.19545)
Supplement: Supplementary file 1 [file oncotarget-08-69983-s001.pdf]

Linc-ROR induces epithelial-to-mesenchymal transition in ovarian cancer by increasing Wnt/β-catenin signaling

SUPPLEMENTARY MATERIALS

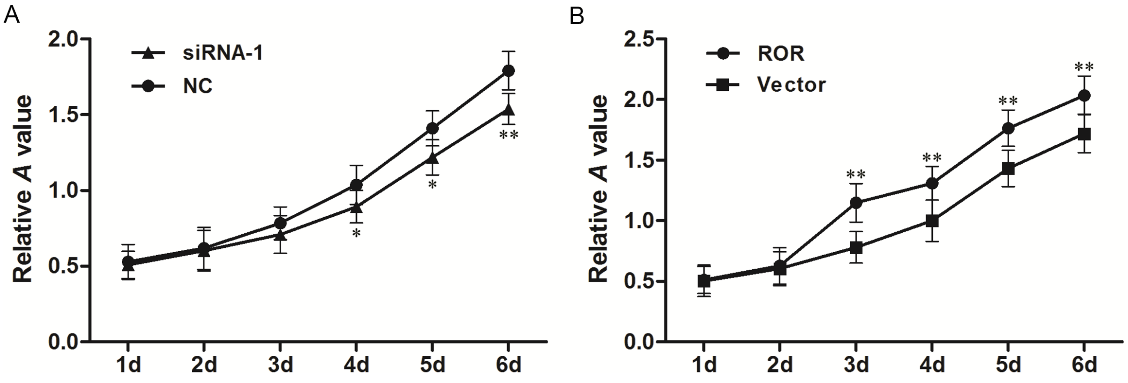

Supplementary Figure 1: Effects of linc-ROR on the proliferation of A2780 cells. Knockdown of linc-ROR reduces the proliferative capacity of A2780 cells **(A)**. Overexpression of linc-ROR promotes the proliferative capacity of A2780 cells **(B)**. *P<0.05; **P<0.01.

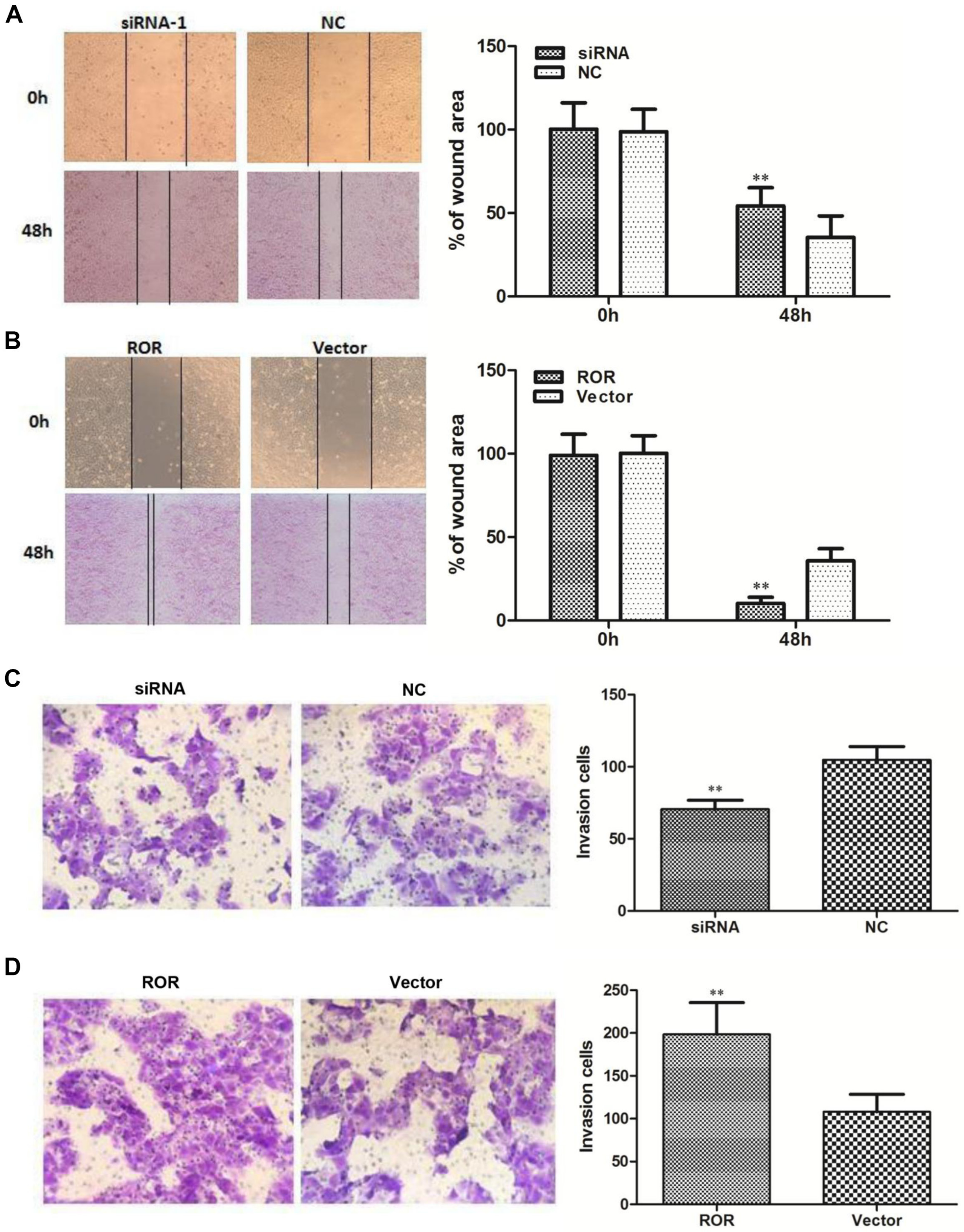

Supplementary Figure 2: Effects of linc-ROR on the migration and invasion of A2780 cells. Linc-ROR would promote the migratory and invasive abilities of A2780 cells. **(A)** and **(B)** Wound healing assay results. **(C)** and **(D)** Transwell results. ** P<0.01.
